# Study on the Adsorption Mechanism of Atrazine by Sesame Hull Biochar/Sepiolite Composite Material

**DOI:** 10.3390/toxics14010038

**Published:** 2025-12-29

**Authors:** Hongyou Wan, Qiuye Yu, Luqi Yang, Shihao Liu, Yan Zhao, Dezheng Chang, Xinru Li

**Affiliations:** 1 School of Ecology and Environment, Zhengzhou University, Zhengzhou 450001, China; yqy5032025@163.com (Q.Y.); ylq3042284309@163.com (L.Y.); lixinru126@outlook.com (X.L.); 2 Henan International Joint Laboratory of Intelligent Water Treatment System, Jiyuan 454650, China; 3 Henan Metallurgical Research Institute Co., Ltd., Zhengzhou 450053, China; liushihao@hnas.ac.cn (S.L.); duojinxing@126.com (Y.Z.); changdezheng@hnas.ac.cn (D.C.)

**Keywords:** sesame straw biochar, sepiolite, atrazine, adsorption mechanism

## Abstract

Atrazine (ATZ), a typical triazine herbicide with a long half-life and recalcitrant biodegradation, contaminates water and soil, necessitating efficient removal technologies. Conventional adsorbents have limited capacity and stability, while sesame straw-derived biochar realizes agricultural waste recycling and provides an efficient, economical, and eco-friendly adsorbent. Sepiolite, a natural mineral with a unique fibrous structure and a high specific surface area, has attracted widespread attention. Therefore, in this work, the agricultural waste of sesame hulls and sepiolite were used as precursors to prepare a composite material of sesame hull biochar/sepiolite (KNPB) through co-mixing heat treatment, followed by sodium hydroxide activation and pyrolysis. The results showed that, under the conditions of an adsorbent dosage of 3 g/L, pH of 6.8, and an adsorption time of 360 min, the removal rate of 3 mg/L ATZ by KNPB was 89.14%. Reusability experiments further demonstrated that KNPB has the potential for practical application in water treatment. Additionally, by integrating adsorption kinetics and isotherm analysis with a suite of characterization results from BET, FTIR, and XPS, the adsorption mechanism of KNPB for ATZ was further clarified to be primarily based on pore-filling, π–π interactions, and hydrogen bonding. This study not only provides a new idea for the resource utilization of waste sesame straw, but also provides scientific guidance for the solution of atrazine pollution, which has important environmental and economic significance.

## 1. Introduction

Atrazine (ATZ) is the most widely used herbicide among triazine herbicides [1]. It is used for weed removal in corn, sorghum, and in orchards and other places because of its low cost and obvious weed suppression effect [2]. However, only part of atrazine in the soil can act on weeds, and the rest remains in the soil, resulting in the accumulation of atrazine in the soil. Atrazine has the characteristics of a stable structure, high migration rate, long half-life, and difficult biodegradation [3]. It will enter surface water and groundwater with surface runoff, leaching and other effects, causing serious pollution to soil and water. Although atrazine is effective in weed control, its potential environmental risks cannot be ignored, so measures need to be taken to reduce atrazine residues in the environment.

At present, the methods to remove ATZ from water include adsorption, microbial method and advanced oxidation technology [4,5,6]. Among them, the adsorption method is widely used because of its simple, economic and efficient advantages and environmental friendliness. Biochar is a porous carbon material prepared by the process of biomass pyrolysis [7]. Biomass sources are diverse, including plants, crop waste, municipal waste, and sludge [8,9,10,11]. The remarkable characteristics of biochar include its large specific surface area, rich pore structure, and oxygen-containing functional groups. These characteristics make biochar show a wide range of application potential in many fields such as adsorption, catalysis and soil improvement. Especially in recent years, sesame straw as a renewable biomass resource has attracted increasing attention in its comprehensive utilization. As a lignocellulosic biomass, sesame seed hull is a potential source of cellulose, hemicellulose and lignin. Studies have shown that biochar prepared from sesame straw shows excellent performance in the competitive adsorption of heavy metals [12]. Xu et al. prepared biochar (WS-1LB) from lignin extracted from sesame straw with acid [13]. The experimental results showed that the biochar of lignin extracted from white sesame straw with acid showed the highest benzopyrene (BaP) adsorption efficiency of 91.44%. Ma et al. successfully prepared a three-dimensional stacked tubular mesoporous biomass carbon material by activating and carbonizing the discarded sesame capsule shell, and applied it to high-performance supercapacitors, showing excellent electrochemical performance [14]. In conclusion, the preparation of biochar from sesame straw not only has significant advantages in environmental and resource utilization, but also shows broad application prospects in many fields.

However, the adsorption capacity of single biochar is usually difficult to meet the requirements of efficient removal of organic pollutants in the environment. In order to improve the adsorption capacity of biochar and reduce the preparation cost, the materials composed of clay minerals and biomass-based biochar have attracted much attention in the field of water treatment. Among them, sepiolite is a magnesium-rich fibrous silicate clay mineral. Its crystal structure is mainly composed of two continuous silicon--oxygen tetrahedra and a discontinuous magnesium--oxygen octahedron in the middle [15]. The structure of sepiolite has rich adsorption sites and surface activities [16]. Studies have shown that the adsorption removal rate of bisphenol A and oxytetracycline by sepiolite modified with brominated hexadecyl trimethyl amine (CTAB) can reach 100% [17]. Sepiolite can also act as a cocatalyst to promote electron transfer in deep oxidation reactions. Jin et al. prepared layered double hydroxide (LDH) acidified sepiolite and simultaneously photocatalytically degraded methyl orange and methylene blue [18]. The composite material exhibited the highest photocatalytic activity under visible light irradiation. Therefore, this study aims to develop a sesame straw biochar/sepiolite composite material by utilizing the rich pore structure of sesame straw biochar and the unique fibrous structure and high specific surface area of sepiolite. The goal is to leverage the porous structure of biochar to support clay minerals, while the combination of clay minerals and biochar helps enhance the stability and performance of the material.

## 2. Materials and Methods

### 2.1. Materials

The sesame capsule shell was from Jia County (33°48′–34°10′ N,113°0′–113°24′ E), Henan, China. Atrazine (C_8_H_14_ClN_5_, ≥99%) and was procured from Aladdin Reagent Co., Ltd. (Shanghai, China). Methanol (MeOH, ≥99%) and NaOH were purchased from Kemiou Chemical Reagent Co., Ltd. (Tianjin, China). HCl was purchased from Chemical Reagent Co., Ltd. (Nanjing, China). All experimental water was pure water.

### 2.2. Preparation of KNPB

The sesame capsule was washed with pure water. The cleaned sesame capsules were dried in a 70 °C oven, then crushed with a multi-functional crusher. The sepiolite and sesame capsule shells were weighed according to the mass ratio of 1:4, where 200 mL of deionized water were added to each sample, followed by ultrasonic dispersion for 1 h, and then mixed the suspensions. The mixed suspension was placed in a constant temperature heating magnetic mixer and stirred at 80 °C for 5 h. The mixture was dried in a 70 °C blower drying oven for 12 h, and then the composite material was ground to powder. The composite material and 1 M NaOH were mixed in a ratio of 1:10 and stirred on a magnetic stirrer for 14 h. After drying, the material was pyrolyzed in a tube furnace in N_2_ atmosphere at 500 °C, 5 °C/min for 2 h. The pyrolyzed composite material was named KNPB. And, the sesame capsule biochar was named KBC.

### 2.3. Characterization Method of KNPB

The surface morphology of KNPB was analyzed using a Scanning Electron Microscope (SEM, Helios G4 CX, Thermo Fisher Scientific, Waltham, MA, USA). Surface area analysis of the KNPB samples was performed using a Brunauer–Emmett–Teller (N_2_-BET) analyzer (ASAP2460, Micromeritics Instrument Corp, Norcross, GA, USA). Fourier-Transform Infrared spectroscopy (FTIR) (Nicolet iS20, Thermo Fisher Scientific, Waltham, MA, USA) was used to determine the functional groups present in KNPB. The crystal structure of KNPB was observed using X-ray diffraction (XRD, Bruker D8 Advance, Bruker Corporation, Karlsruhe, BW, Germany). Surface functional groups of KNPB was studied by X-ray photoelectron spectroscopy (XPS, K-Alpha, Thermo Fisher Scientific, Waltham, MA, USA). The thermal stability of KNPB was studied by thermogravimetric analyzer (TG, TGA/DSC1, Mettler Toledo, Greifensee, Zurich, Switzerland).

### 2.4. Batch Adsorption Experiment

Isotherms experiments were carried out using 50 mL of ATZ solution (1–20 mg/L) with 0.15 g of KNPB, and the mixtures were shaken at 150 rpm for 6 h. Kinetic adsorption experiments were performed using 50 mL of 3 mg/L ATZ solution with 0.15 g of KNPB, sampled at different time intervals (5, 15, 30, 60, 90, 180, 360 min). Here, 0.15 g of KNPB was added to 50 mL of 3 mg/L ATZ solution. The mixture was shaken under 150 rpm for 6 h at 25 °C. To investigate the effect of pH on adsorption, 0.1 M HCl and 0.1 M NaOH were used to adjust the initial pH to levels of 3, 5, 6.8, 8, 10, respectively. Additionally, the impact of temperature on adsorption was studied at 15 °C, 25 °C, and 35 °C. The effects of KNPB dosage (0.5–4.0 g/L), initial concentration of ATZ (1–20 mg/L) on the ATZ removal efficiency of KNPB were also explored. After the adsorption kinetics experiment, the material was washed with MeOH and pure water in a ratio of 1:1, and recovered by high-speed centrifuge. The material was dried at 70 °C. After drying, repeated adsorption experiments were carried out, and a total of 3 cycles of experiments were carried out and sampled for analysis.

The mixed solutions were filtered through a needle filter (organic system, 0.45 μm) for further testing. All the batch sorption experiments were conducted in triplicate. And the supernatants were analyzed by high-performance liquid chromatography (HPLC, Agilent Technologies 1260 Infinity II, Agilent Technologies, Santa Clara, CA, USA). The adsorption capacity and removal rate of ATZ in this experiment were calculated by Equations (1) and (2):

(1) $$Q=\frac{(C_{0}-C_{t})V}{m}$$

(2) $$R=\frac{\left(C_{0}-C_{t}\right)}{C_{0}}\times 100\%$$

where Q (mg/g) is the amount of equilibrium adsorption; C_0_ and C_t_ are the concentrations of ATZ at time zero (initial concentration) and t, respectively; V (L) is the ATZ volume; m (g) is the amount of KNPB; and R (%) is the removal efficiency.

### 2.5. Analysis of Atrazine

The ATZ concentration in this experiment was analyzed by HPLC using a C_18_ column (250 mm × 4.6 mm), sample input volume of 10 µL, column temperature of 35 °C, and detection wavelength of 222 nm. The mobile phase was MeOH and pure water in the ratio of 7:3 (*v*:*v*). The flow rate was 0.8 mL/min. And, the mean experimental data were plotted using Origin 2021.

## 3. Results and Discussion

### 3.1. Characterization of KNPB

The physicochemical properties of biochar and composite materials are shown in Table 1. Compared with KBC, the O content of KNPB was reduced, while the N content increased after Sepiolite was doped on the surface of biochar. According to (Table 1), the specific surface area of KNPB (36.80 m^2^/g) was 10.3 times larger than that of single KBC (3.23 m^2^/g). Moreover, the average pore size of KNPB (17.60 nm) was larger than that of KBC (13.82 nm), and the total pore volume (0.07 cm^3^/g) was much larger than that of KBC (0.0097 cm^3^/g), indicating that NaOH activation significantly improved the specific surface area of the composite material. This may be because NaOH dissolves the ash in the pores of biochar and effectively clears the pores inside and outside the biochar, resulting in an increase in the specific surface area and an increase in the average pore size [19].

**Figure 1:**
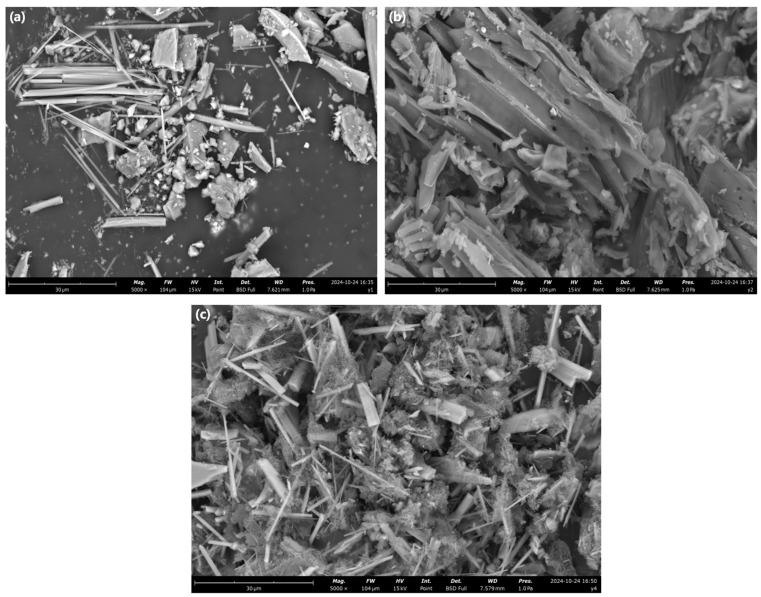
Scanning electron microscope images. (**a**) Sep; (**b**) KBC; (**c**) KNPB.

The SEM image of clay mineral Sep is shown in Figure 1a. It can be seen that Sep exhibited rod-like structures of varying lengths [20], with a smooth and densely scattered surface, accompanied by minor impurity minerals. Figure 1b,c shows the morphology images of KBC and KNPB, respectively. In KBC, a flake-like structure is observed, with pores of varying sizes distributed throughout. The flake-layer structure of KNPB presents irregular crack-like pores that interweave with the rod-like structure of sepiolite. The porous structure provides abundant diffusion pathways for ATZ molecules, thereby accelerating adsorption. Moreover, the surface roughness of KNPB is significantly increased, and the number of pores has also increased. This is because the biochar treated with alkali is eroded on its surface, forming a rougher surface structure [21].

**Figure 2:**
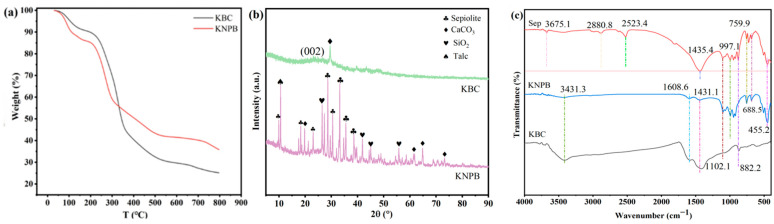
(**a**) Thermogravimetric curves of KNPB and KBC; (**b**) XRD of KNPB and KBC; (**c**) FTIR of KNPB, KBC, and Sep. The absorption peak at 3431.3 cm^−1^ arises from the stretching vibration of the -OH group. An absorption peak is observed at 1680.6 cm^−1^, confirming the presence of carboxyl groups in the material. The absorption peak around 1431.1 cm^−1^ can be attributed to the C=C vibration of aromatic rings, which facilitates the adsorption of aromatic pollutants by biochar through π-π interactions.

The thermogravimetric analysis curve of KNPB for when the temperature increased from 30 °C to 800 °C, is shown in Figure 2a. The weight loss of KNPB was mainly concentrated in two stages, among which the weight loss of 89.1 °C was reduced due to the desorption of adsorbed water. The weightlessness at 357 °C was obvious because of the decomposition of volatile organic compounds [22] and the removal of oxygen-containing functional groups as well as the removal of water of crystallization in sepiolite, this has led to a significant decline in quality at this stage. Alkali modification etches the biochar surface, disrupts part of the fragile carbon skeleton, and introduces more labile oxygen-containing functional groups—resulting in the increased loss of these functional groups. Meanwhile, it promotes graphitization and carbon-skeleton reconstruction, thereby enhancing the thermal stability of the core structure. Additionally, compared to KBC, KNPB exhibited lower mass loss, indicating better thermal stability, Compared with KBC, the DTG curve of KNPB slows down, indicating that the activation of NaOH enhances the thermal stability of biochar.

The crystal structure of KBC and KNPB is shown in (Figure 2b). A wide and narrow diffraction peak corresponding to the (002) plane was present near 23° in KBC. The diffraction peaks indicated that graphite and disordered graphite planes were the main characteristics of biochar, and the presence of graphite structure can enhance the adsorption of pollutants through π-π action [23,24]. In addition, the XRD of KBC showed that the biochar contained calcium carbonate, which may be related to the composition of sesame capsules. As for KNPB, 2θ = 26.6° was the characteristic peak of SiO_2_, and the diffraction peak appearing at 2θ = 33° matched the characteristic peak (080) of the sepiolite cubic crystal cell (JCPDS No. 13-0595) [25]. The XRD results of KNPB showed the successful doping of SiO_2_, CaCO_3_ and talc mineral crystals, indicating that KNPB does not alter the crystal structure of biochar and sepiolite, but rather integrates the mineral crystal composition of the two materials. So it can be known that sepiolite is successfully doped on KBC.

The FTIR of KNPB is shown in Figure 2c. The absorption peak of KNPB at 3431 cm^−1^ was attributed to the stretching vibration of -OH groups [26]. Carboxyl groups exhibit asymmetric and symmetric stretching vibrations of the O-C=O bonds in the region of 1650–1560 cm^−1^ [27]. The absorption peak at 1608 cm^−1^ was due to the stretching vibration of the C=O bond, indicating the presence of carboxyl groups in KNPB. The absorption peak at 1431 cm^−1^ was attributed to the stretching vibration of the C=C double bonds in aromatic rings [28]. This is favorable for the adsorption of aromatic pollutants by biochar through π-π interactions [29]. The absorption peaks near 994.9 cm^−1^ and 754.4 cm^−1^ in Sep are due to the stretching vibrations of the Si-O groups in the Si-O-Si bonds [30]. The absorption peak at 451 cm^−1^ can be attributed to the symmetric or asymmetric stretching vibrations of the Si-O-Mg bonds on the surface [31]. The characteristic peaks of Sep at 759 cm^−1^, 688 cm^−1^, and 455 cm^−1^ were observed in KNPB, which indicates that Sep has been successfully doped onto KBC.

### 3.2. The Effect of Sorbent Type on ATZ Adsorption

**Figure 3:**
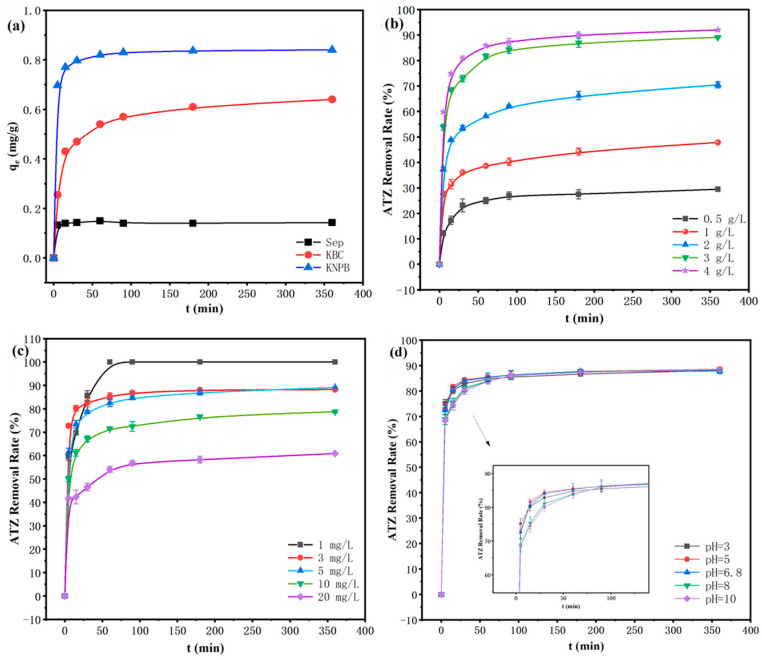
(**a**) Adsorption of ATZ by different adsorbents; (**b**) Effect of KNPB dosage on ATZ removal efficiency; (**c**) Effect of initial concentration of ATZ on removal efficiency; (**d**) PH influence on ATZ removal efficiency. Conditions: t = 360 min; V_ATZ_ = 50 mL; C_ATZ_ = 3 mg/L; dosage (KNPB) = 3 g/L.

As depicted in Figure 3a, the adsorption performance of the three materials for ATZ showed significant differences. The composite material (KNPB) exhibited the fastest adsorption rate in the initial stage and reached equilibrium in a relatively short time, with an adsorption capacity of 0.84 mg/g, demonstrating the best adsorption performance. A single KBC followed, with an adsorption capacity of around 0.6 mg/g and a slower adsorption rate compared to KNPB. Sep had the poorest adsorption performance, with an adsorption capacity of only about 0.2 mg/g, possibly due to its high internal crystallization water and adsorption water content, which results in a low adsorption capacity. This indicated that the composite KNPB combined the rich pore structure of KBC and the unique crystal structure of Sep, which significantly enhances the adsorption capacity of ATZ.

#### 3.2.1. Effect of KNPB Dosage on Adsorption of ATZ

The effect of the addition amount (0.5 g/L~4.0 g/L) on the adsorption of ATZ was studied by adding different masses of KNPB into a 50 mL ATZ solution of 3 mg/L, as shown in Figure 3b. As can be seen from Figure 3b, with the increase in KNPB dosage, the removal rate of ATZ also increases. When the dosage is increased from 0.5 g/L to 3 g/L, the removal rate of KNPB increased from 27.97% to 88.91%. However, when the dosage of KNPB continued to increase to 4 g/L, the removal efficiency of ATZ was 90.66%, and the increase was not significant compared with that at 3 g/L. This is attributed to the increased number of available adsorption sites provided by the higher KNPB dosage, which enhances the removal efficiency. Notably, the adsorption capacity (q_e_) of KNPB for ATZ increases with the equilibrium concentration of ATZ (up to the full saturation of active sites), in line with fundamental adsorption principles. However, increasing the sorbent dose reduces the equilibrium concentration of ATZ in the solution, thereby limiting the further improvement of adsorption capacity. This explains why the ATZ removal rate only slightly increases to 90.66% when the KNPB dosage is further increased to 4 g/L (compared with 3 g/L). It is speculated that, when the KNPB dosage reaches a certain threshold (3 g/L in this study), the ATZ concentration in the solution becomes relatively low, and the adsorption sites tend to be saturated. Further increasing the dosage has a limited effect on improving the removal efficiency due to the reduced equilibrium concentration. Therefore, 3 g/L was selected as the optimal KNPB dosage for subsequent experiments.

#### 3.2.2. Effect of Initial Concentration on Adsorption of ATZ

The effect of initial concentration on the adsorption of ATZ was investigated by adding 3 g/L KNPB to the solution of ATZ (50 mL) with different initial concentrations (1 mg/L, 3 mg/L, 5 mg/L, 10 mg/L, 20 mg/L), as shown in Figure 3c. As shown in Figure 3c, KNPB can completely remove 1 mg/L of ATZ within 6 h. When the ATZ concentration increases from 3 mg/L to 20 mg/L, the removal efficiency of ATZ decreased from 89.13% to 62.83%. This is because, when the ATZ concentration is low (such as 1 mg/L), the adsorption sites on the surface of the adsorbent are sufficient to adsorb the ATZ molecules in the solution, thus achieving a higher removal efficiency. However, with the increase in ATZ concentration, the adsorption sites on the surface of the adsorbent were gradually occupied, resulting in a decrease in the number of available adsorption sites, thus limiting the removal efficiency.

#### 3.2.3. pH Effects on ATZ

As shown in Figure 3d, 3 g/L KNPB was added to 3 mg/L ATZ (50 mL) solution, and pH was adjusted to 3, 5, 6.8, 8, and 10 to investigate the effect of pH on the adsorption of ATZ. In the range of the pH value from 3 to 10, KNPB showed a good removal effect on ATZ with a removal rate of more than 88% within 360 min. However, within the first 100 min of reaction, there was a significant difference in the adsorption rate of ATZ, which was significantly higher under acidic conditions than under alkaline conditions. Figure S1 shows the Zeta potential of KNPB is close to 0 under acidic conditions, which is conducive to the adsorption of ATZ. However, under alkaline conditions, the Zeta potential value of KNPB is lower than that under acidic conditions, and it is not conducive to adsorption due to electrostatic repulsion. The pH experiment results show that KNPB can efficiently remove ATZ within a wide pH range and has good adaptability to wastewater with different pH levels. The results show that KNPB can effectively remove ATZ in a wide range of pH and has good adaptability to wastewater with different pH values.

#### 3.2.4. Sorption Kinetics

**Figure 4:**
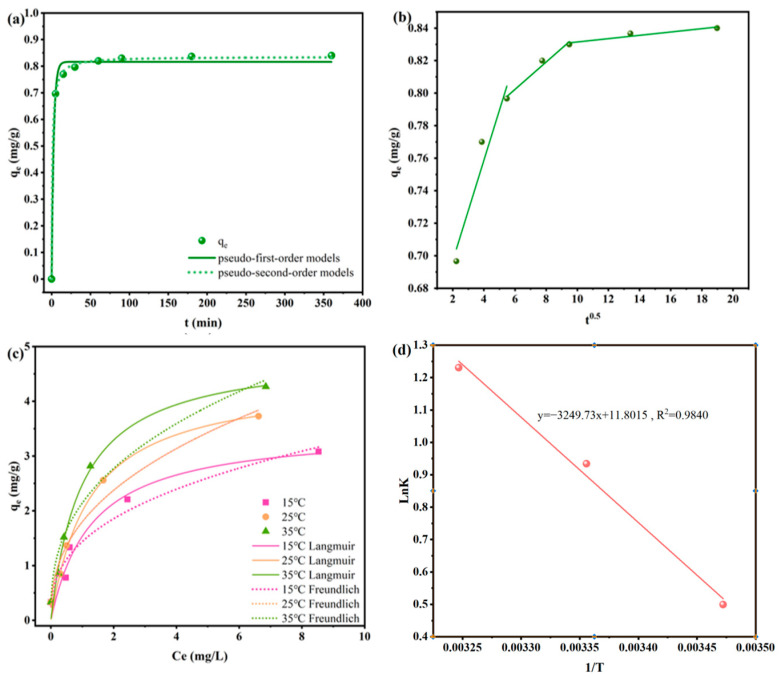
(**a**) Kinetics of adsorption of ATZ by KNPB; (**b**) Particle internal diffusion model fitting; (**c**) Equilibrium adsorption fitting of ATZ by KNPB; (**d**) Thermodynamic study of KNPB. Conditions: t = 360 min; V_ATZ_ = 50 mL; C_ATZ_ = 3 mg/L; dosage (KNPB) = 3 g/L; pH = 6.8; T = 25 °C.

The adsorption kinetics were fitted by quasi-first-order kinetics and quasi-second -order kinetics, respectively. The results are shown in Figure 4a, and the fitting parameters of the adsorption kinetics are shown in Table 2. Figure 4a shows the adsorption amount of KNPB on ATZ with the adsorption time (0~360 min). In the first 5 min, the adsorption amount of KNPB on ATZ increased rapidly, and the adsorption increases slowly in 5~60 min, and gradually reached an equilibrium state from 60~360 min. As can be seen in Table 2, the correlation coefficients R^2^ of the quasi-first-order and quasi-second-order kinetic models of KNPB adsorption on ATZ are 0.9929 and 0.9992, respectively, indicating that both the quasi-first-order and quasi-second-order kinetic models can well describe the adsorption process of KNPB on ATZ. It was shown that, in addition to physical adsorption, chemical adsorption also played a leading role in the adsorption of ATZ by KNPB [32].

To further study the adsorption and diffusion mechanism, a particle internal diffusion model was used to fit the adsorption kinetics, as shown in Figure 4b. The fitting parameters of the particle internal diffusion model were presented in Table 3. The linear relationship of the adsorption on ATZ did not pass through the origin, indicating that internal diffusion was not the sole limiting factor but was determined by multiple factors including surface adsorption and internal diffusion. As shown in (Figure 4b), the adsorption of ATZ by KNPB can be divided into three stages: The first stage was liquid film diffusion, where the adsorbent surface had multiple adsorption sites, allowing ATZ to rapidly adsorb onto the adsorbent surface, resulting in the fastest diffusion rate and the highest K value; the second stage was internal particle diffusion, where ATZ diffused inward within the adsorbent, with a slower diffusion rate and a significant decreased in K value; the third stage saw a reduction in the concentration of ATZ in the solution, leading to increased diffusion resistance. At this point, the diffusion rate became more moderate, and the K value continues to decrease until equilibrium was reached.

#### 3.2.5. Adsorption Isotherm and Adsorption Thermodynamics

The isothermal adsorption was fitted by Langmuir and Freundlich model equations, respectively, and the results are shown in (Figure 4c). The fitting parameters are shown in (Table 4). The R^2^ value of Langmuir model fitting was 0.9587~0.9949, while the R^2^ value of Freundlich model fitting was 0.9064~0.9300, indicating that compared with the Freundlich model, the adsorption of ATZ by KNPB was more consistent with the Langmuir model, indicating that the adsorption of KNPB was a single molecular layer adsorption. When the temperature increased from 15 °C to 35 °C, the theoretical maximum adsorption capacity increased from 3.50 mg/g to 4.9 mg/g, which was consistent with the thermodynamic results of adsorption heat absorption. Although the Freundlich model was slightly lower than the Langmuir model, the correlation coefficient was also above 0.90. Meanwhile, the K_f_ value increased with the increase in temperature, indicating that the adsorption amount of KNPB increased with the increase in temperature, which was consistent with the results of isotherm adsorption experiment.

The thermodynamic parameters of KNPB adsorption ATZ were shown in (Table 5). With the increase in temperature, ∆G was negative and decreased with the increase in temperature, indicating that the adsorption process was spontaneous. Meanwhile, both ∆H and ∆S were positive, indicating that the adsorption was an endothermic reaction.

### 3.3. Reusability

**Figure 5:**
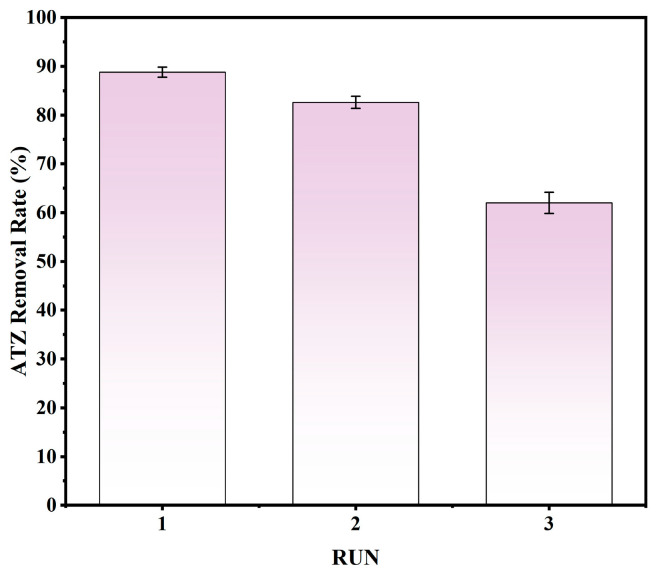
The recycling capacity of KNPB.

Repeatability and stability tests are among the factors that determine whether a material has practical application potential. After each adsorption experiment, the composite material was recovered by centrifugation, washed with water and MeOH several times, dried and then the next adsorption experiment was carried out. The results are shown in Figure 5. In the first two cycles, KNPB had a good adsorption effect on ATZ, and the removal rate of ATZ was more than 80%. In the third cycle, the removal rate of ATZ was still 62.02%, indicating that KNPB had a good reusability.

### 3.4. Adsorption Mechanism

The physicochemical properties of KNPB before and after ATZ adsorption were compared by Zeta potential, FTIR, and XPS to reveal the adsorption mechanism of ATZ on KNPB. According to Table 1, the specific surface area of KNPB was 10 times larger than that of KBC, the total pore volume and average pore size were also improved under the modification of NaOH. These results indicated that the adsorption process involves pore filling, mainly mesoporous adsorption. The removal ability of ATZ by KNPB under different pH had no significant effect (Figure 3d). Zeta potential showed that KNPB was negatively charged in a wide range of pH (Figure S1), which indicated that the influence of electrostatic adsorption on adsorption capacity may not be obvious. According to the analysis of adsorption kinetics and isotherms (Figure 4a,c), both quasi-first-order and quasi-second-order kinetic models can effectively describe the adsorption process of KNPB on ATZ. This indicated that, in addition to physical adsorption, chemical adsorption also played a dominant role in the adsorption of KNPB on ATZ. The adsorption of KNPB on ATZ was more consistent with the Langmuir model, suggesting that the adsorption of KNPB was monolayer adsorption.

The FTIR results (Figure S2) showed that the absorption peak of KNPB at 3431 cm^−1^ was generated by the stretching vibration of -OH. The amplitude of the -OH peak of KNPB after adsorption increased, indicating that ATZ had an interaction with the hydroxyl group. The absorption peak at 1608 cm^−1^ was generated by the stretching vibration of C=O. After adsorption, the amplitude of C=O increased, which may be due to chemical bonds or complexation reactions were formed between the adsorbent and C=O functional group. The absorption peak at 1431 cm^−1^ was produced by the vibration of C=C double bond of aromatic ring [28]. After adsorption, the intensity of C=C absorption peak was weakened, which suggested that during the adsorption process, C=C functional group may react with the adsorbed substance or transform into other functional groups. The absorption peaks near 759 cm^−1^, 688 cm^−1^, and 455 cm^−1^ were generated by the stretching vibration of Si-O group and Si-O-Mg bond in Si-O-Si, whilst the modified material still retained the characteristic peak of Sep.

The XPS spectra of KNPB before and after adsorption of ATZ were analyzed. As can be seen from (Figure S3a), the main characteristic peaks of KNPB appeared in O 1s and C 1s, and the presence of N, Mg, Na, Ca, and Si is also detected. After adsorption of ATZ, a new Cl 2p peak appeared at 199.8 eV, indicating that ATZ had been successfully adsorbed on the surface of KNPB. At the same time, the C-C content of KNPB after the adsorption of ATZ increased from 61.6% to 63.03%, and the C-O content slightly increased from 23.0% to 23.6% (Figure S3b), indicating that the graphitization structure of KNPB was enhanced after adsorption of ATZ. The content of C=O peak decreased from 50.6% to 39.79% (Figure S3c), indicating that C=O was involved in the adsorption of ATZ. After the adsorption of ATZ, the content of pyrrole nitrogen (398.8 eV) increased, indicating that more nitrogen-containing groups related to ATZ may be introduced in the adsorption process (Figure S3d). This indicated that an π-π interaction between ATZ and KNPB may occur, thus promoting the adsorption process.

**Figure 6:**
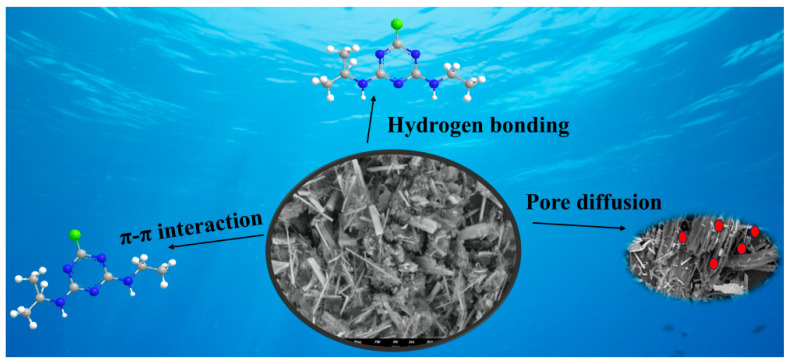
Adsorption mechanism of ATZ by KNPB.

Some studies have shown that the π-π interaction between aromatic rings in organic compounds and adsorbents was an important factor affecting the adsorption process [33]. According to FTIR, the aromatic ring C=C functional group of KNPB changed before and after adsorption, so it was speculated that π-π interaction may have occurred with ATZ. Hydrogen bonding was also one of the main adsorption mechanisms [34], and the oxygen-containing functional groups on KNPB can form hydrogen bonding with atrazine. In general, the entire adsorption process of ATZ on KNPB involved a variety of mechanisms and interactions, including pore filling, π-π interaction, and hydrogen bonding (Figure 6).

## 4. Conclusions

In this work, using the agricultural waste of sesame hulls and sepiolite as precursors, a composite material of sesame hull biochar/sepiolite (KNPB) was prepared through co-mixing heat treatment followed by sodium hydroxide activation and pyrolysis. The aim was to explore a more efficient, economical, and environmentally friendly adsorbent for the effective removal of atrazine (ATZ). In this study, the effects of various adsorption factors on the adsorption performance of KNPB for ATZ were investigated. By comparing the adsorption of atrazine by different adsorbents, the composite material KNPB exhibits superior adsorption performance. The results showed that under the conditions of an adsorbent dosage of 3 g/L, pH of 6.8, and an adsorption time of 360 min, the removal rate of 3 mg/L ATZ by KNPB was 89.14%. Furthermore, the reusability experiments indicated that KNPB had the potential for practical application in water treatment. Combining adsorption kinetics and isotherm analysis with a series of characterization results from BET, FTIR, and XPS, the adsorption mechanism of KNPB for ATZ was further clarified to be primarily based on pore-filling, π-π interactions, and hydrogen bonding. This study not only provides a new approach for the resourceful utilization of waste sesame stalks but also offers theoretical and data support for the development of high-performance adsorbents through experimental research. It will help promote the efficient utilization of agricultural waste and provide scientific guidance for solving atrazine pollution problems, holding significant environmental and economic importance.

## Figures and Tables

**Table 1:** Physicochemical properties of KNPB.

Materials	C (%)	O (%)	N (%)	Specific Surface Area (m^2^/g)	Pore Volume (cm^3^/g)	Average Pore Size (nm)
KBC	78.59	20.09	1.32	3.23	0.0097	13.82
KNPB	78.86	18.76	2.38	36.80	0.0700	17.60

**Table 2:** Adsorption kinetics fitting data.

Material	q_e_, exp (mg/g)	Pseudo-First-Order Model			Pseudo-Second-Order Model		
		q_e_ (mg/g)	K_1_ (min^−1^)	R^2^	q_e_ (mg/g)	K_2_ (g/(mg·min))	R^2^
KNPB	0.83	0.8164	0.3789	0.9929	0.8357	1.1342	0.9992

**Table 3:** Particle internal diffusion fitting data.

Material	Period	Particle Internal Diffusion		
		C	K	R^2^
KNPB	Stage Ι	0.6351	0.0309	0.8704
	Stage ΙΙ	0.7518	0.0084	0.9546
	Stage III	0.8212	0.0010	0.8377

**Table 4:** Isothermal adsorption fitting data.

Material	T	Langmuir			Freundlich		
	°C	Q_m_ (mg/g)	K_L_	R^2^	K_f_	1/n	R^2^
KNPB	15	3.5068	0.7780	0.9587	1.4385	0.3676	0.9167
	25	4.4235	0.8194	0.9949	1.7987	0.4011	0.9300
	35	4.9024	1.0218	0.9941	2.1422	0.3729	0.9064

**Table 5:** Adsorption thermodynamic data.

T (K)	∆G (J·mol^−1^)	∆H (kJ·mol^−1^)	∆S (J·mol^−1^)
288	−1195.70	27.01	98.11
298	−2314.81		
308	−3151.74		

## Data Availability

The original contributions presented in this study are included in the article/Supplementary Materials. Further inquiries can be directed to the corresponding authors.

## Supplementary Materials

The following supporting information can be downloaded at https://www.mdpi.com/article/10.3390/toxics14010038/s1, Figure S1: Zeta potential diagram of KNPB; Figure S2: FTIR before and after KNPB adsorption of ATZ; Figure S3: XPS spectra of KNPB before and after ATZ adsorption. (a) Full spectrum analysis; (b) C 1s; (c) O 1s; (d) N 1s.

## Abbreviations

The following abbreviations are used in this manuscript.

ATZ	Atrazine
KNPB	A composite material of sesame hull biochar/sepiolite
KBC	Sesame capsule biochar
