# Supplementary material for: Study on the Adsorption Mechanism of Atrazine by Sesame Hull Biochar/Sepiolite Composite Material
Source: Toxics. 2025 Dec 29;14(1):38. doi: 10.3390/toxics14010038 (PMC12845623; doi:10.3390/toxics14010038)
Supplement: Supplementary file 1 [file toxics-14-00038-s001.zip › toxics-4022472-supplementary.pdf]

Supplementary Information

Study on the Adsorption Mechanism of Atrazine by Sesame Hull Biochar/Sepiolite Composite Material

Hongyou Wan [1,*], Qiuye Yu [1], Luqi Yang [1,2], Shihao Liu [3], Yan Zhao [3], Dezheng Chang [3] and Xinru Li [1,2]

[1] School of Ecology and Environment, Zhengzhou University, Zhengzhou 450001, China; yqy5032025@163.com (Q.Y.); ylq3042284309@163.com (L.Y.); lixinru126@outlook.com (X.L.)
[2] Henan International Joint Laboratory of Intelligent Water Treatment System, Jiyuan 454650, China
[3] Henan Metallurgical Research Institute Co., Ltd., Zhengzhou 450053, China; liushihao@hnas.ac.cn (S.L.); duojinxing@126.com (Y.Z.); changdezheng@hnas.ac.cn (D.C.)
* Correspondence: hywan@zzu.edu.cn

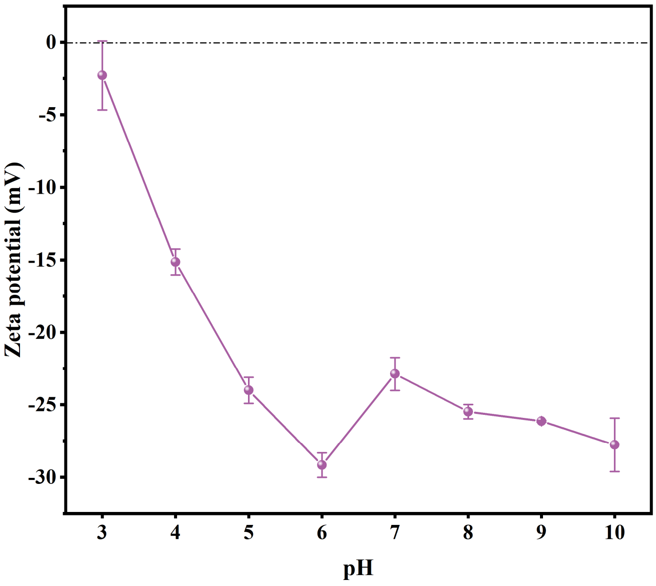

Figure S1. Zeta potential diagram of KNPB

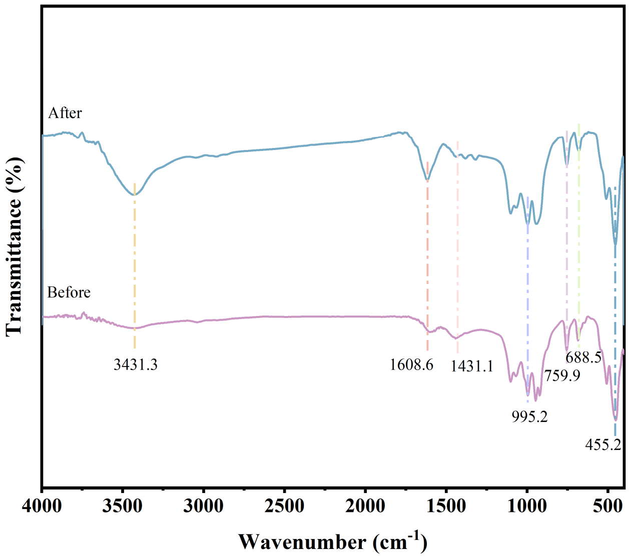

Figure S2. FTIR before and after KNPB adsorption of ATZ

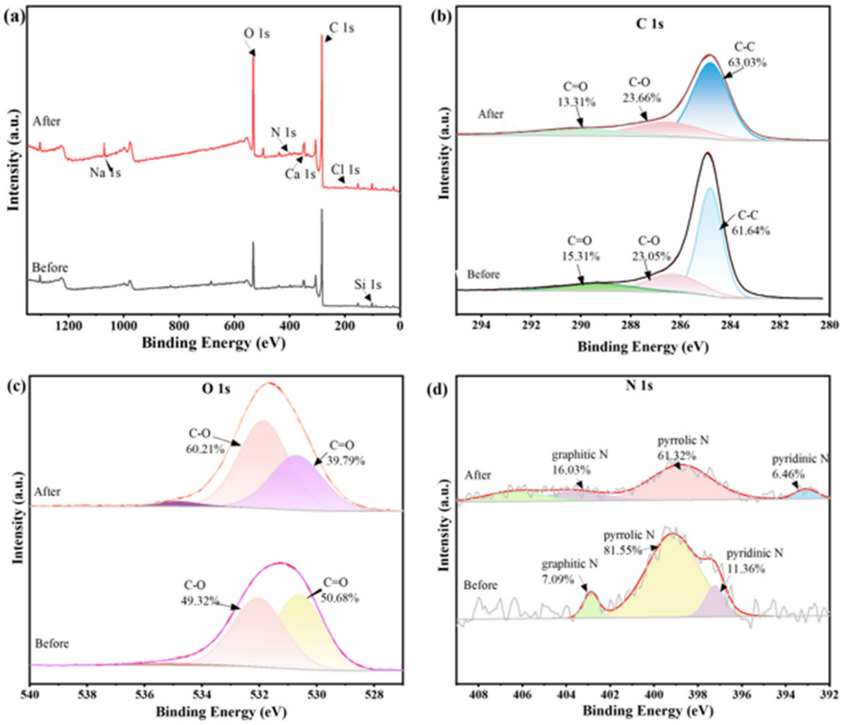

Figure S3. XPS spectra of KNPB before and after ATZ adsorption.(a)Full spectrum analysis; (b) C 1s; (c) O 1s; (d) N 1s
